# Associations between attention-deficit/hyperactivity disorder and allergic diseases: a two-sample Mendelian randomization study

**DOI:** 10.3389/fpsyt.2023.1185088

**Published:** 2023-07-05

**Authors:** Xiangyu Zhang, Runlong Zhang, Yuanfeng Zhang, Tao Lu

**Affiliations:** ^1^School of Life Sciences, Beijing University of Chinese Medicine, Beijing, China; ^2^School of Acupuncture-Moxibustion and Tuina, Beijing University of Chinese Medicine, Beijing, China; ^3^China Science and Technology Development Center for Chinese Medicine, Beijing, China

**Keywords:** attention-deficit/hyperactivity disorder, allergic diseases, Mendelian randomization, allergic asthma, allergic rhinitis, pollen allergy, allergic urticaria, allergic conjunctivitis

## Abstract

**Background:**

In some observational studies, attention-deficit/hyperactivity disorder has been linked to allergic diseases, but the findings are debatable. This study aimed to determine whether attention-deficit/hyperactivity disorder (ADHD) is causally related to allergic asthma, allergic rhinitis, pollen allergy, allergic urticaria, and allergic conjunctivitis using the two-sample Mendelian Randomization (MR) approach.

**Methods:**

We did a two-sample Mendelian randomization (MR) study, which chose single nucleotide polymorphisms (SNPs) that are highly associated with attention-deficit/hyperactivity disorder (ADHD) levels from the Psychiatric Genomics Consortium (PGC) on 20,183 cases and 35,191 controls as our instruments. Outcomes datasets included genome-wide association study (GWAS) meta-analysis (*n* = 1,415,804). The summary statistics of outcome data were obtained from the FinnGen datasets including allergic asthma (10,877 cases and 180,942 controls), allergic rhinitis (8,430 cases and 298,829 controls), pollen allergy (4555cases and 301,734 controls), allergic urticaria (1792 cases and 299,491 controls) and allergic conjunctivitis (15,567 cases and 293,587 controls). Inverse variance weighted, MR-Egger, weighted median, were used to estimate the causal association between ADHD and allergic diseases. Cochran’s Q test was used to quantify the heterogeneity of instrumental variables. MR-Egger intercept test, leave-one-out analysis, and the funnel plot were all used in sensitivity analyses.

**Results:**

The Mendelian randomization (MR) analysis indicated that ADHD in inverse variance weighted [odds ratio (OR) = 1.0612; 95% confidence interval (CI):1.0192–1.1049; *p* = 0.0039] lightly increased the risk of allergic asthma. In MR sensitivity analyses of the weighted median, a similar association was found. But no evidence for an effect of ADHD on allergic asthma risk was found in additional methods: MR-Egger (OR = 0.9592, 95% CI: 0.8384–1.0974, *p* = 0.5457), and weighted median (OR: =1.0341, 95% CI: 0.9785–1.0929, *p* = 0.2330). Also, no strong evidence for an effect of ADHD on other allergic diseases (allergic rhinitis, pollen allergy, allergic urticaria, and allergic conjunctivitis) incidence was found using the inverse variance weighted (IVW) method, weighted median method, and MR-Egger regression.

**Conclusion:**

Although several studies have found a link between ADHD and allergic diseases, our findings do not support that ADHD could increase allergic diseases incidence. Randomized controlled trials or Mendelian randomization studies with larger samples are still needed to draw more precise conclusions.

## 1. Introduction

Attention-deficit/hyperactivity disorder (ADHD), which is a common and disruptive disorder that affects children, adolescents, and adults (1, 2), is characterized by excessive inattention and/or hyperactivity, as a lifespan neurodevelopmental condition with specific criteria for children and adults description in the Diagnostic and Statistical Manual of Mental Disorders (second edition; DSM-5). The symptoms and deficits of ADHD usually show up as early as preschool and often continue into adulthood (3). A follow-up study of children with ADHD found that those who had ADHD throughout childhood had lower educational attainment, job performance, and emotional control as adults than those who did not (4). Meanwhile, adults diagnosed with ADHD have higher mortality rate ratios than those diagnosed in childhood and adolescence (2).

The relationship between attention-deficit/hyperactivity disorder and allergic diseases such as allergic asthma has received much attention in recent years. Whether ADHD increases the risk of allergic disease remains controversial (5). And previous studies are primarily based on relatively small case–control studies subject to selection bias, reverse causation, and confounding; meta-analyses report considerable heterogeneity of results. RCTs are expensive and time-consuming; therefore, alternative methods to strengthen causal inference from observational data could help guide whether such trials are potentially warranted. Mendelian randomization (MR) is a causal inference method that, if certain assumptions are met, can overcome issues of reverse causation and confounding that weaken inferences from observational analyses by using genetic variants as instrumental variables (6). The fundamental principle of MR is that genetic instruments capable of predicting the degree of a modifiable exposure should be causally linked to the exposure-related result. A valid genetic instrument must make three assumptions: (i) it is causally related to the exposure, (ii) it is independent of confounders, and (iii) it is only associated with the outcome through the exposure. We, therefore, aimed to apply a two-sample MR approach to investigate whether ADHD is related to five allergic diseases (allergic asthma, allergic rhinitis, pollen allergy, allergic urticaria, and allergic conjunctivitis).

## 2. Methods

### 2.1. Study design

**Figure 1:**
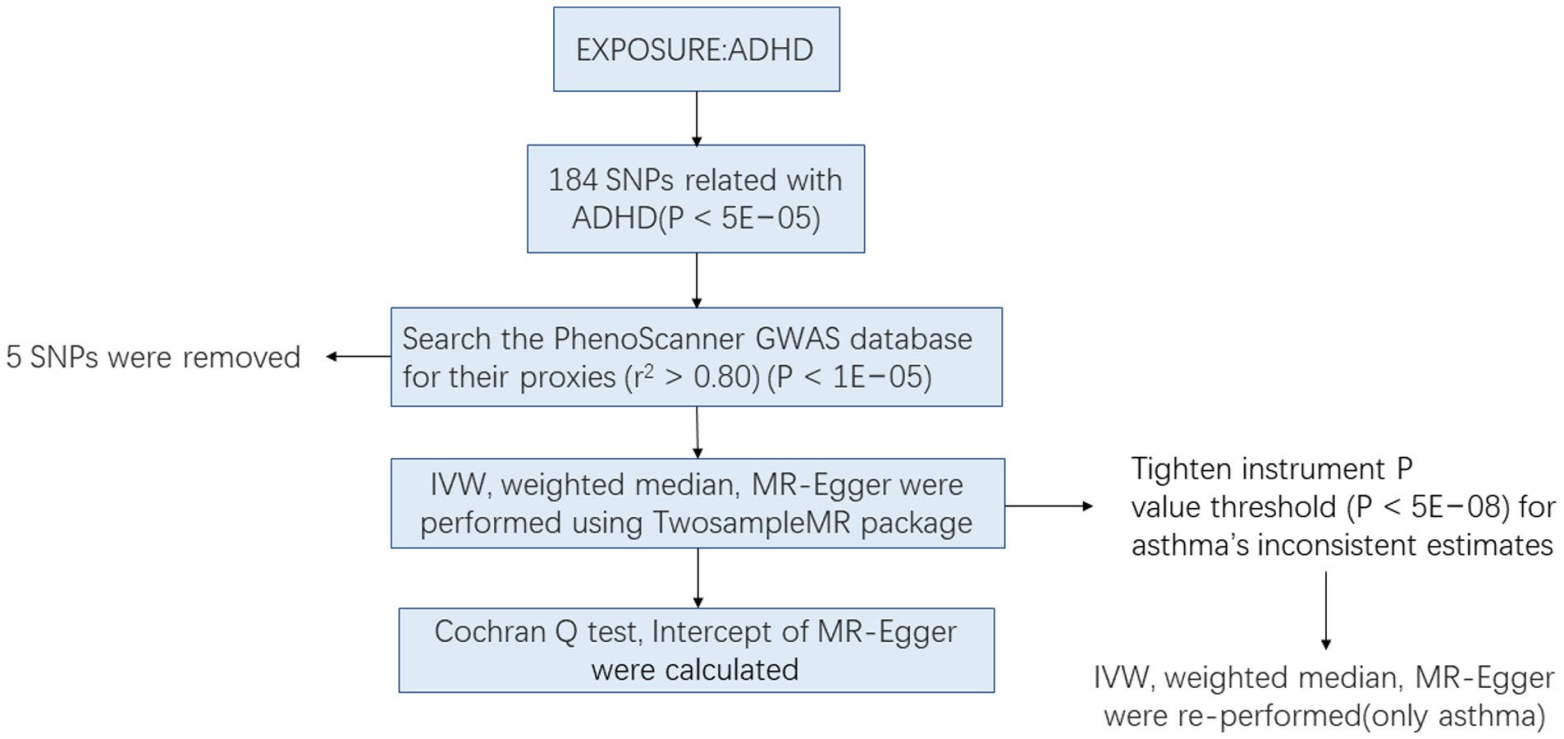
Workflow of Mendelian randomization study.

In this study, we did a two-sample Mendelian randomization study, using deidentified summary-level data that were publicly available (Figure 1). Overall age and gender information were not calculable from the summary-level GWAS results. Information about the data sources and sample sizes used in this study are summarized in Table 1. Ethical approval was obtained in all original studies.

**Table 1:** Attention-deficit/hyperactivity disorder and allergic diseases genetic summary data sources.

Category	Trait	Sample size	*n* case	*n* control	Population
Exposure	ADHD*	55,374	20,183	35,191	European, North American, Chinese
Outcomes	allergic asthma*	191,819	10,877	180,942	European
	allergic rhinitis*	307,259	8,430	298,829	European
	pollen allergy*	306,289	4,555	301,734	European
	allergic urticaria*	301,283	1792	299,491	European
	allergic conjunctivitis*	309,154	15,567	293,587	European

ADHD, attention-deficit/hyperactivity disorder.

*Available from: https://pgc.unc.edu/for-researchers/download-results/; https://www.finngen.fi/en/access_results.

### 2.2. Data sources and instruments

GWAS for attention-deficit/hyperactivity disorder were obtained from the meta-analysis published to data for Psychiatric Genomics Consortium (PGC) (7). The study included 20,183 individuals diagnosed with ADHD and 35,191 controls. These data were collected from 12 cohorts (Table 1) including a population-based cohort of 14,584 cases and 22,492 controls from Denmark collected by the Lundbeck Foundation Initiative for Integrative Psychiatric Research (iPSYCH), and 11 European, North American, and Chinese cohorts aggregated by the Psychiatric Genomics Consortium (PGC). ADHD cases in iPSYCH were identified from the national Psychiatric Central Research Register and diagnosed by psychiatrists at a psychiatric hospital using ICD10 codes (F90.0) (7). 184 single-nucleotide polymorphisms (SNPs) were reported significantly related to ADHD(*p* < 5 × 10^−5^, linkage disequilibrium. [LD] r2 < 0.001, Clumping distance>10,000 kb). Details of the 184 SNPs are listed in Supplementary Table S1. These SNPs explained 6.73% of the variability in attention-deficit/hyperactivity disorder. The F-statistics, which was greater than the expected value of 10, was 3729.4 (8), showing that the instruments had a high potential for predicting attention-deficit/hyperactivity disorder (9).

Outcomes datasets included GWAS summary statistics of allergic asthma (10,877 cases and 180,942 controls), allergic rhinitis (8,430 cases and 298,829 controls), pollen allergy (4555cases and 301,734 controls), and allergic urticaria (1792 cases and 299491controls) allergic conjunctivitis (15,567 cases and 293,587 controls) from the FinnGen datasets. For FinnGen, we used the data from the R7 which was updated on 2022.6.1. The detailed description is provided on the FinnGen research project website.^1^

### 2.3. Statistical analysis

In MR analysis, inverse variance weighted (IVW) method is often used as the main MR method because of its strong ability to identify the effect. However, the IVW method requires that instrumental variables influence the outcome only through exposure in the study. Even though known confounding SNPs were excluded from this study, there are still many unknown confounding factors that could lead to gene polymorphism and bias effect size estimates. Among the other two supplementary Mendelian randomization methods, MR-Egger regression is not as strict as the use premise of IVW. At the same time, this method can not only detect the pleiotropy but also correct the pleiotropy bias. The core of this method is that the intercept term is used to measure the average pleiotropy between instrumental variables in the weighted linear regression, and the slope is an unbiased estimate of the causal effect (10).

Because the median is more robust than the remote value, in order to have a more reliable MR estimate, the Weighted Median method is adopted to complement the MR-egger regression. In this method, the weighted median estimate is the 50% weighted percentile, with the MR estimates ranked in order of inverse variance from smallest to largest. If more than 50% of the weight comes from valid SNPs, then the method can be considered to provide an unbiased estimate of the effect of the MR.

Due to different sources of data, MR analysis may have heterogeneity, resulting in a bias in the estimation of the causal effect. Consequently, in this study, the main IVW analysis method and MR-Egger regression were tested for heterogeneity. If the *p*-value is above 0.05, the instrumental variables included in the analysis are not heterogeneous.

Pleiotropy included vertical and horizontal pleiotropy. Vertical pleiotropy means that a genetic variation can only affect one trait by affecting another trait, which is the basis of MR. To evaluate the causal relationship between two diseases. Horizontal pleiotropy means that genetic variation can affect another trait through multiple independent pathways. In this paper, the pleiotropy test mainly refers to horizontal pleiotropy. One of the assumptions of MR analysis is that instrumental variables can only have an effect on outcomes through exposure. It is therefore necessary to test for gene pleiotropy in the causal inference between exposure and outcome. MR-Egger regression analysis can be used to assess the bias caused by pleiotropy, and its regression intercept can be used to assess the magnitude of the pleiotropy. The likelihood of pleiotropy is lower the closer the intercept is to 0. The *p*-value of the pleiotropy test was used to measure the presence of pleiotropy in the analysis. If *p* > 0.05, pleiotropy was considered weak in the causal analysis, and its effect was ignored.

In the present study, the leave-one-out approach was also used for sensitivity analysis. This means that each relevant SNP was removed in turn and the combined effect of the remaining SNPs was calculated to assess the influence of each SNP on the disease for diseases with a *p*-value of less than 0.05 in the IVW method and which passed the heterogeneity test and the gene pleiotropy test.

Mendelian randomization analyses were performed using R version 4.2.1 with the Two Sample MR package (R Project for Statistical Computing). To ensure that the impact estimates of each SNP on ADHD and the result related to the same effect allele, all ADHD-associated SNPs were harmonized with the outcome data in FinnGen (Supplementary Table S2). For each instrument SNP, we searched the PhenoScanner GWAS database (version 2; http://phenoscanner.medschl.cam.ac.uk) for their proxies (r2 > 0.80) to assess any previous associations (*p* < 1E−05) with potential confounding traits (11) (Table 2). We then evaluated the effects of manually removing these SNPs from the MR analysis to rule out potential pleiotropic effects. We employed different MR techniques to generate MR estimates between ADHD and allergic diseases after harmonizing the impact alleles across the GWASs of ADHD and allergic diseases, notably, the inverse variance weighted (IVW), weighted median, and MR-Egger.

**Table 2:** 5 SNPs were removed, which might induce pleiotropy issues given its association with risk factors for allergic diseases.

SNP	Pos (hg19)	A1	A2	Trait	Type
rs111900779	chr12:112379095	A	G	Eosinophil count	Diseases and traits
rs17531412	chr1:44182244	A	G	Maternal smoking around birth	Diseases and traits
rs62260755	chr3:49898318	C	G	Past tobacco smoking	Diseases and traits
rs696825	chr9:86583076	T	C	Eosinophil percentage of granulocytes	Diseases and traits
rs7087891	chr10:8792629	A	T	No blood clot, bronchitis, emphysema, asthma, rhinitis, eczema or allergy diagnosed by doctor. Asthma Self-reported asthma	Diseases and traits

Sensitivity analysis has been essential in MR investigations for detecting underlying pleiotropy. Cochrane’s Q statistics were used to quantify the heterogeneity. The MR-Egger intercept (12) and methods were used to detect horizontal pleiotropy. Leave-one-out analysis was also undertaken to see whether a single SNP was leading or biasing the MR estimate.

## 3. Results

**Figure 2:**
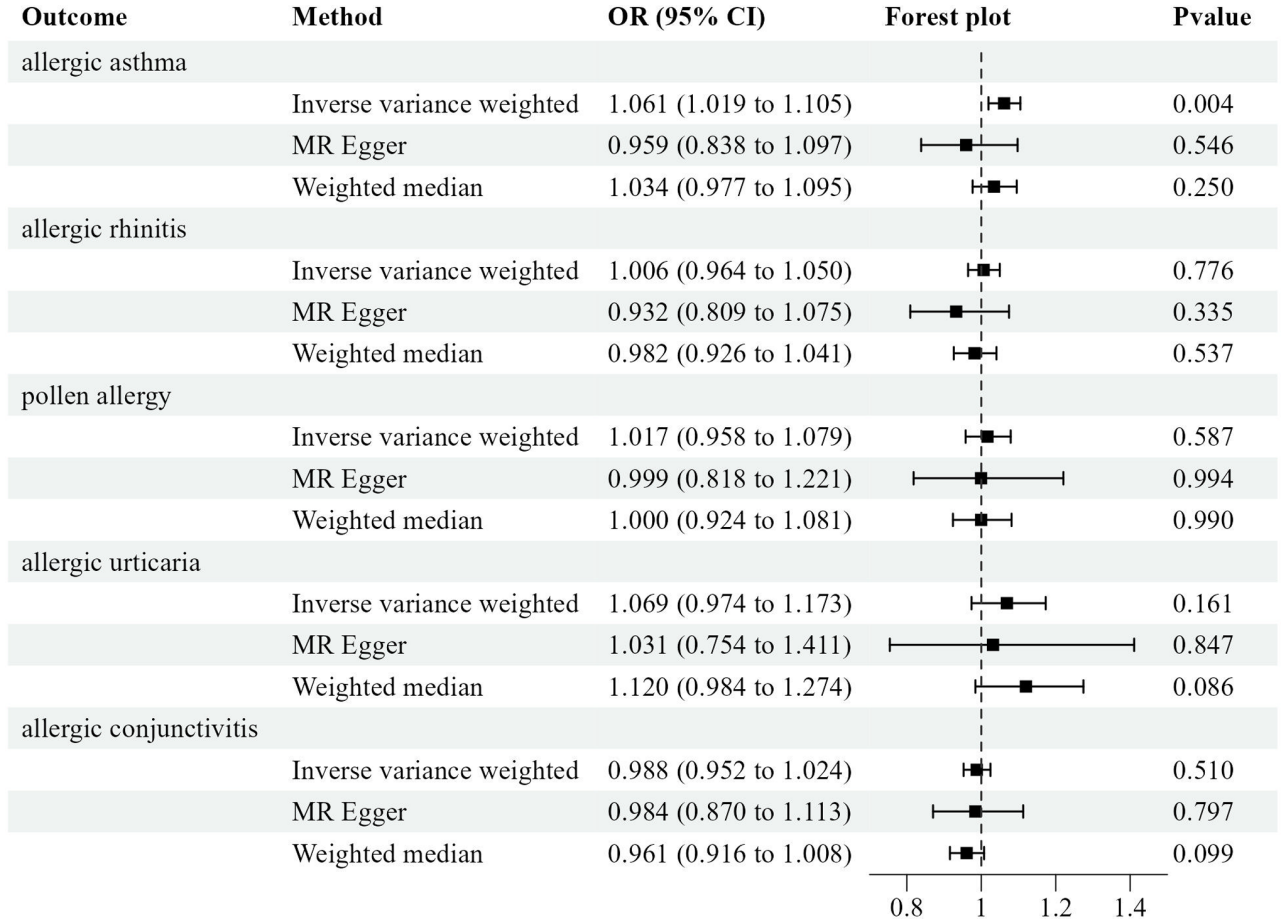
Odds ratio plot for five allergic diseases.

With the IVW method, ADHD increased the risk for allergic asthma significantly (OR = 1.0612, 95% CI = 1.0192–1.1049, *p* = 0.0039), while opposing results were observed using the MR-Egger approach (OR = 0.9593, 95%CI =0.8384–1.0974, *p* = 0.5457). Meanwhile, the weighted median (OR = 1.0342, 95%CI =0.9766–1.0952, *p* = 0.2500) approach garnered comparable risk estimates, though the association was not statistically significant (Table 3; Figure 2; Supplementary Figure S1).

**Table 3:** MR Results for the causal effect of ADHD on five allergic diseases.

Method	No. of SNPs	MR analysis		Heterogeneity test		MR-Egger intercept *p*
		OR (95%)	*p*	Cochran’s Q	*p*	
ADHD on allergic asthma						
IVW	138	1.0612146	0.003911085	166.6077	0.04330413	–
MR-Egger	138	0.9592501	0.545714756	163.7489	0.05263483	0.1256657
Weighted median	138	1.0341863	0.250041500	–	–	–
ADHD on allergic rhinitis						
IVW	138	1.0061724	0.7756071	146.4394	0.2750124	–
MR-Egger	138	0.9324609	0.3354811	145.1440	0.2800504	0.2725305
Weighted median	138	0.9817702	0.5311091	–	–	–
ADHD on pollen allergy						
IVW	138	1.0166279	0.5867152	157.4762	0.1112198	–
MR-Egger	138	0.9992600	0.9942229	157.4400	0.1007526	0.8598359
Weighted median	138	0.9995059	0.9906810	–	–	–
ADHD on allergic urticaria						
IVW	138	1.0687520	0.1614441	156.0481	0.1268954	–
MR-Egger	138	1.0314480	0.8465849	155.9858	0.1156452	0.8160901
Weighted median	138	1.1198770	0.08590793	–	–	–
ADHD on allergic conjunctivitis						
IVW	138	0.9878218	0.50997907	195.8353	0.0007281909	–
MR-Egger	138	0.9840203	0.79739867	195.8293	0.0005977502	0.9486676
Weighted median	138	0.9605944	0.09497609	–	–	–

We tightened the instrument *p*-value cut off to 5 × 10^−8^ and employed 9 SNPs as instrument tools because the MR estimations of MR-Egger and IVW were incompatible. The MR estimates became nonsignificant, showing that the genetically predicted increase in ADHD was not associated with an increased risk of allergic asthma (Table 4; Supplementary Table S2).

**Table 4:** MR Results for the causal effect of ADHD on allergic asthma (*p* <5 × 10^−8^).

Method	No. of SNPs	MR analysis		Heterogeneity test		MR-Egger intercept *p*
		OR (95%)	*p*	Cochran’s Q	*p*	
IVW	9	1.037448	0.6565262	0.04410478	15.88171	–
MR-Egger	9	1.275181	0.5351707	0.03376302	15.17975	0.5871734
Weighted median	9	1.150156	0.1007851	–	–	–

IVW, inverse variance weighted; MR, mendelian randomization; OR, odds ratio; SNP, single-nucleotide polymorphism.

Horizontal pleiotropies were not found in the MR-Egger intercept test (*p* = 0.1257), there was no evidence for heterogeneity in the Cochran’s Q test (IVW derived Q statistic = 166.6077; *p* = 0.0433), or leave-one-out analysis (Table 3; Supplementary Figure S1).

The result of IVW indicated that attention-deficit/hyperactivity disorder had no causality on allergic rhinitis (OR = 1.0062, *p* = 0.7756), pollen allergy (OR = 1.0166, *p* = 0.5867), allergic urticaria (OR = 1.0688, *p* = 0.1614), and allergic conjunctivitis (OR = 0.9878, *p* = 0.5100) (Figure 2; Supplementary Figures S2–S5). The Cochran Q-test derived *p*-value was greater than 0.05, suggesting that no evident heterogeneity was identified (Table 3).

## 4. Discussion

In this study, we aimed to use Mendelian randomization to assess whether ADHD affects the incidence of allergic diseases and found no clear evidence supporting the effect of ADHD on the risk of five allergic diseases.

In the MR analysis, the inverse variance weighted (IVW) method is adopted as the main MR Method. At the same time, two other methods were used to test the reliability and stability of the results, namely MR Egger regression and the weighted median method. MR analysis was conducted for ADHD and five allergic diseases in turn. If the above three different MR Models produced similar estimates of causal effect, then the causal relationship between ADHD and allergic diseases was considered stable and reliable. In the results of the IVW analysis, we used *p* < 0.05 to test for significant causality. And combined with the *p*-value of the other two methods to judge.

Meanwhile, the data were tested for heterogeneity and gene pleiotropy. If the *p*-value is greater than 0.05 in the results of the heterogeneity test, it is considered that there is no heterogeneity in the included instrumental variables, and the influence of heterogeneity on the estimation of causal effect can be ignored. If the *p*-value of the gene pleiotropy test <0.05, it is considered that the possibility of gene pleiotropy in the causal analysis is weak, and its influence can be ignored.

In this study, Mendelian randomized analysis was performed on ADHD and allergic asthma, and it was found that ADHD significantly increased the risk of allergic asthma when *p* was taken as IVW method (OR = 1.0612, 95%CI = 1.0192–1.1049, *p* = 0.0039), and MR-Egger (OR = 0.9593, 95%CI =0.8384–1.0974, *p* = 0.5457) and weighted median (OR = 1.0342, 95%CI =0.9766–1.0952, *p* = 0.2500). The results of the three are inconsistent, which cannot prove causality between the two. When selecting MR analysis, the SNP threshold was narrowed to reduce bias. *p* < 5 × 10^−8^ was a significant inclusion condition for the results of association analysis, and *p* values of the three methods were all greater than 0.05. This indicates that there is no significant causal relationship between the two. The causal relationship between ADHD and the other four methods is also the same. Therefore, there is no conclusive evidence that ADHD has a causal relationship with the five allergic diseases.

Our results differ from some previous studies. The neurochemical hypothesis of ADHD and allergic illness was first put out in the late 1980s, hypothesizing that allergic responses lead to an imbalance in the cholinergic/adrenergic activity of the central nervous system, which in certain children results in symptoms of ADHD (13). Since then. The relationship between ADHD and allergic diseases is fraught with controversy. Many studies reveal different points of view. In a controlled trial in 1994, there was no significant difference in the risk of asthma between 140 people with ADHD (aged 6 to 17 years) and controls arguing against the clear pathophysiological connection between the two diseases (5). In 2011, the research compared 594 adult ADHD sufferers to 719 members of the general community in a cross-sectional survey. The information was obtained from 1997 to 2005. Asthma prevalence was noticeably greater in the ADHD patient group as compared to the controls (14).

This conclusion is different from the results of some previous observational studies for the following reasons: (i) Based on the GWAS database, the MR analysis method selects appropriate genetic variants (SNPs) as instrumental variables to effectively avoid the bias of confounding factors such as lifestyle and social environment on disease outcomes, (ii) Similar co-morbid patterns exist for anxiety and mood disorders in both ADHD and asthma (14). It is therefore possible that these other co-morbid diseases are a mediator of the relationship between ADHD and asthma. ADHD alters the dopamine system, which has been implicated in many other psychiatric disorders, such as anxiety and depression, and inhaling dopamine drugs can trigger acute asthma (15). Allergic diseases may be caused by other mental diseases, but there is no direct causal relationship between the two, and (iii) this study uses a large sample MR analysis study, which is different from previous observational studies with small samples and is more convincing. At the same time, the previous studies were basic and cohort studies, which could not clarify causality and chronological order. It has the shortcomings of geographic restrictions.

Our study has several strengths. First, using the Mendelian randomization method, a randomized controlled trial can be simulated. Randomized controlled trials have many advantages such as the highest level of evidence, the ability to minimize various possible biases in the design and implementation of clinical trials, balance confounding factors, and improve the validity of statistical tests. However, the sample size is large, the study period is long, and the study cost is high. Two-sample MR analysis using public data based on genome-wide association studies can overcome the limitations of observational epidemiological studies, which use genetic variation as an instrumental variable for exposure to infer causality between exposure and outcome. Because random assignment of genetic variants occurs at gametogenesis, it is not influenced by environmental factors or disease processes and avoids reverse causality. Second, our findings may provide some reference for the health management of ADHD. Although our findings suggest that there is no clear causal relationship between ADHD and allergic diseases, attention should still be paid to the occurrence of ADHD complicated with allergic diseases, and the risk factors caused by the increased exposure to allergic substances due to the large range of activities of ADHD patients.

However, there are some limitations to the article. First, all GWAS data are mainly from European populations, it cannot be shown to be apply to populations of other ethnicities. Second, ADHD is more common in males and it is difficult to stratify the database statistics by gender, thus it is not possible to assess the role of ADHD on the risk of allergic disease in different age groups by gender. This may lead to biased study results.

## 5. Conclusion

This is the first MR study to investigate the relationship between ADHD and allergic diseases. The study findings do not support the idea that ADHD could increase allergic disease incidence. There was little evidence that ADHD affects allergic diseases. Randomized controlled trials or MR studies with larger samples are still needed to draw more precise conclusions.

## Data availability statement

The original contributions presented in the study are included in the article/Supplementary material, further inquiries can be directed to the corresponding authors.

## Ethics statement

All data are publicly available and are approved by the institutional review committees in their respective studies. Therefore, no further sanction was required.

## Author contributions

XZ designed the study and analyzed the data. XZ and YZ prepared the first draft of the manuscript. RZ assisted in analyzing the data. YZ and TL revised the manuscript for intellectual content. All authors have read the manuscript and approved the final version.

## Funding

This study was supported by start grants from Funds for High-grade, Precision and Advanced (Traditional Chinese Medical Science and Life Science) in Beijing (1000062520573) and the Beijing University of Chinese Medicine Medical Engineer Cooperation Research Project (90020361220009).

## Conflict of interest

The authors declare that the research was conducted in the absence of any commercial or financial relationships that could be construed as a potential conflict of interest.

## Publisher’s note

All claims expressed in this article are solely those of the authors and do not necessarily represent those of their affiliated organizations, or those of the publisher, the editors, and the reviewers. Any product that may be evaluated in this article, or claim that may be made by its manufacturer, is not guaranteed or endorsed by the publisher.

## Footnotes

^1^https://www.finngen.fi/en
