# Supplementary figures and images for: Associations between attention-deficit/hyperactivity disorder and allergic diseases: a two-sample Mendelian randomization study
Source: Front Psychiatry. 2023 Jul 5;14:1185088. doi: 10.3389/fpsyt.2023.1185088 (PMC10356558; doi:10.3389/fpsyt.2023.1185088)

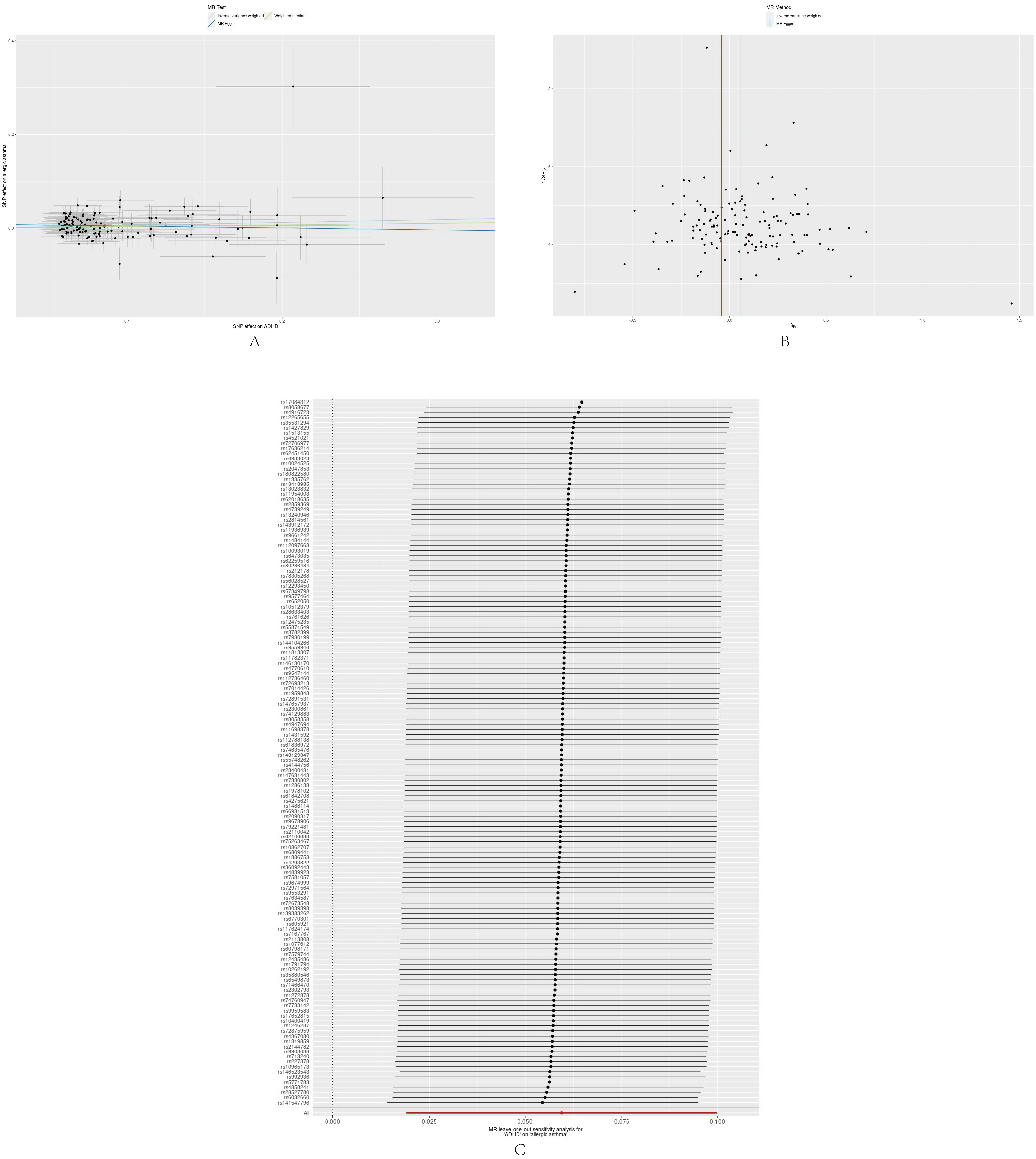

Supplement: Supplementary file 4 [file Image_1.JPEG]

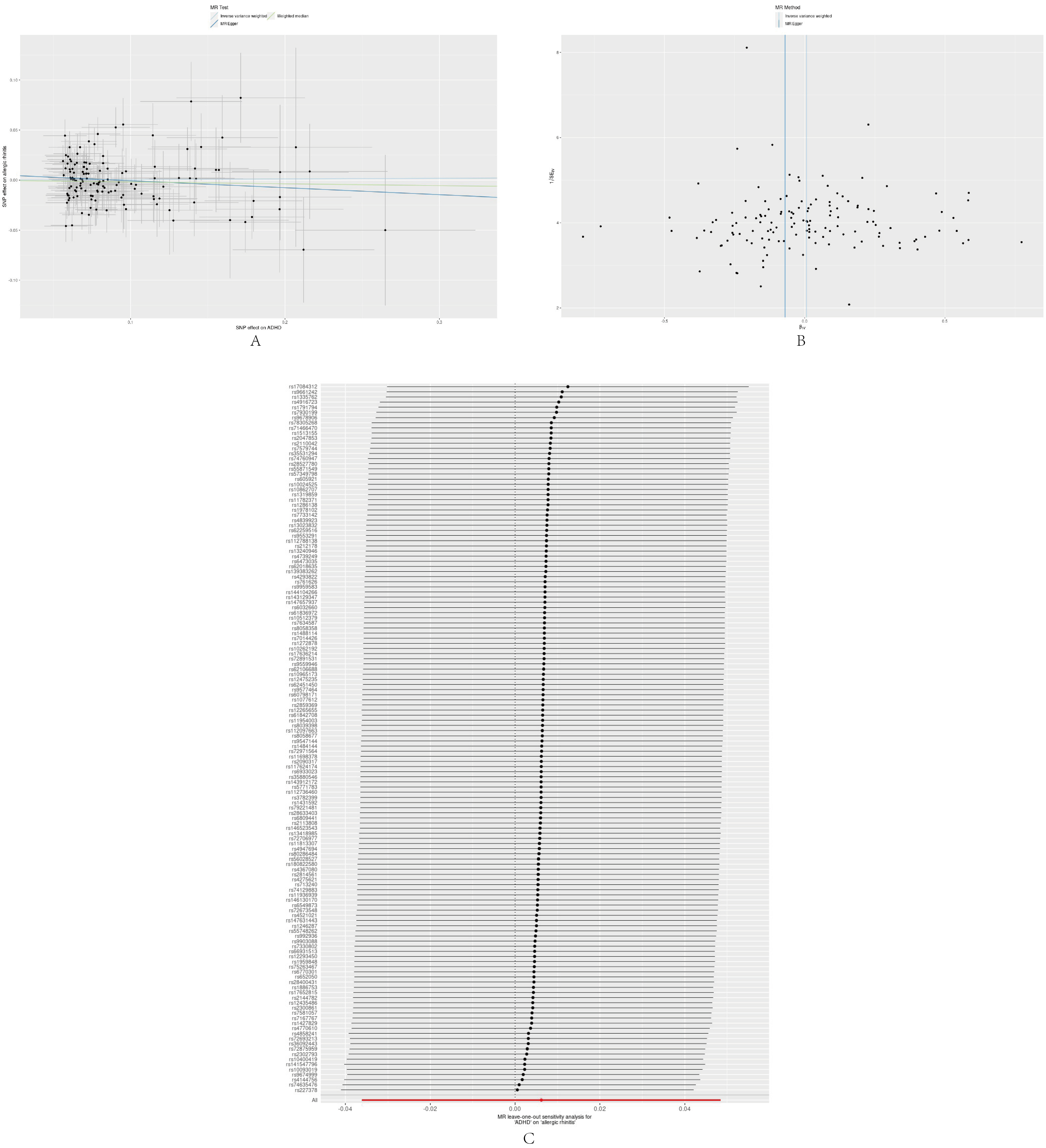

Supplement: Supplementary file 5 [file Image_2.JPEG]

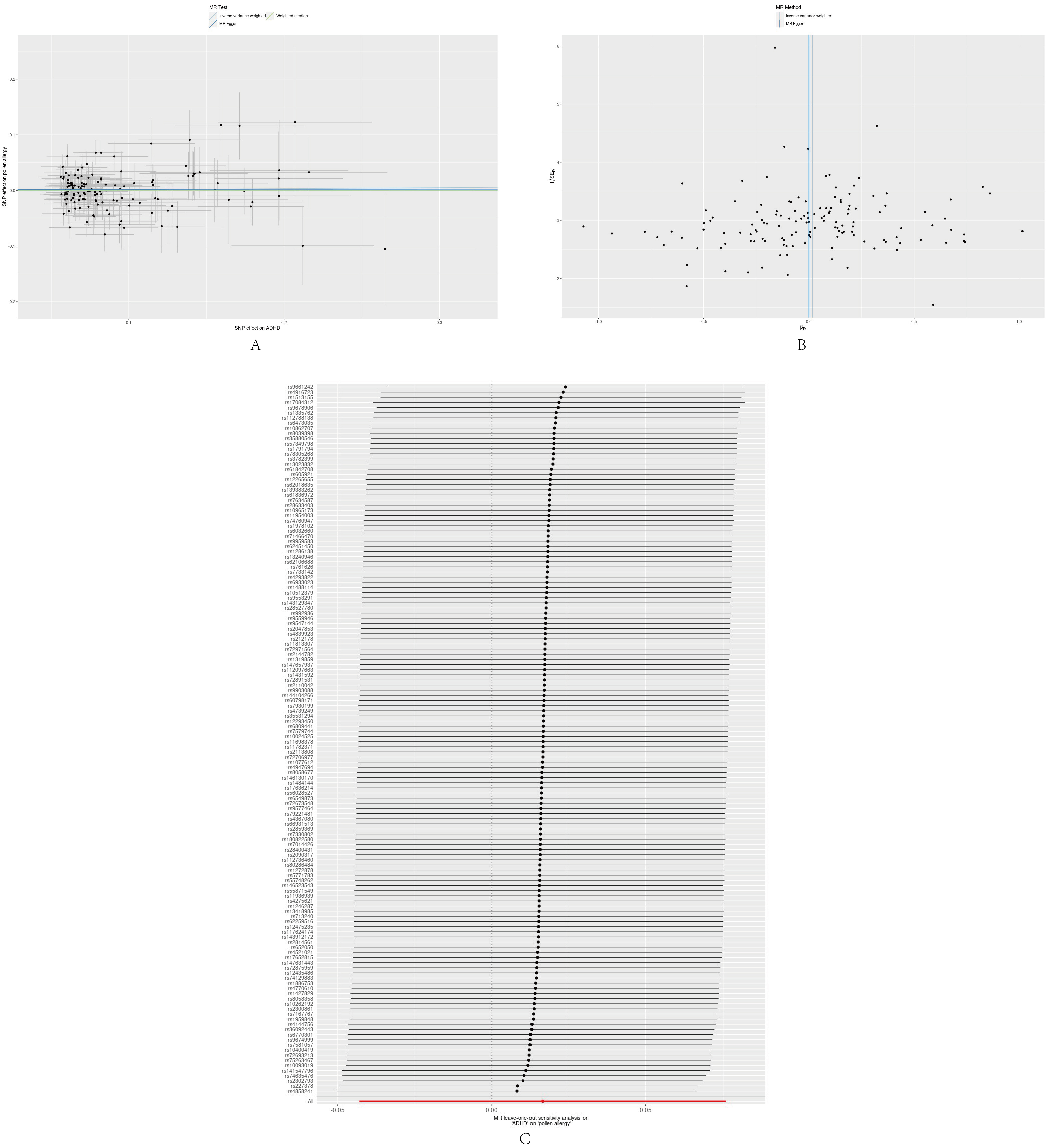

Supplement: Supplementary file 6 [file Image_3.JPEG]

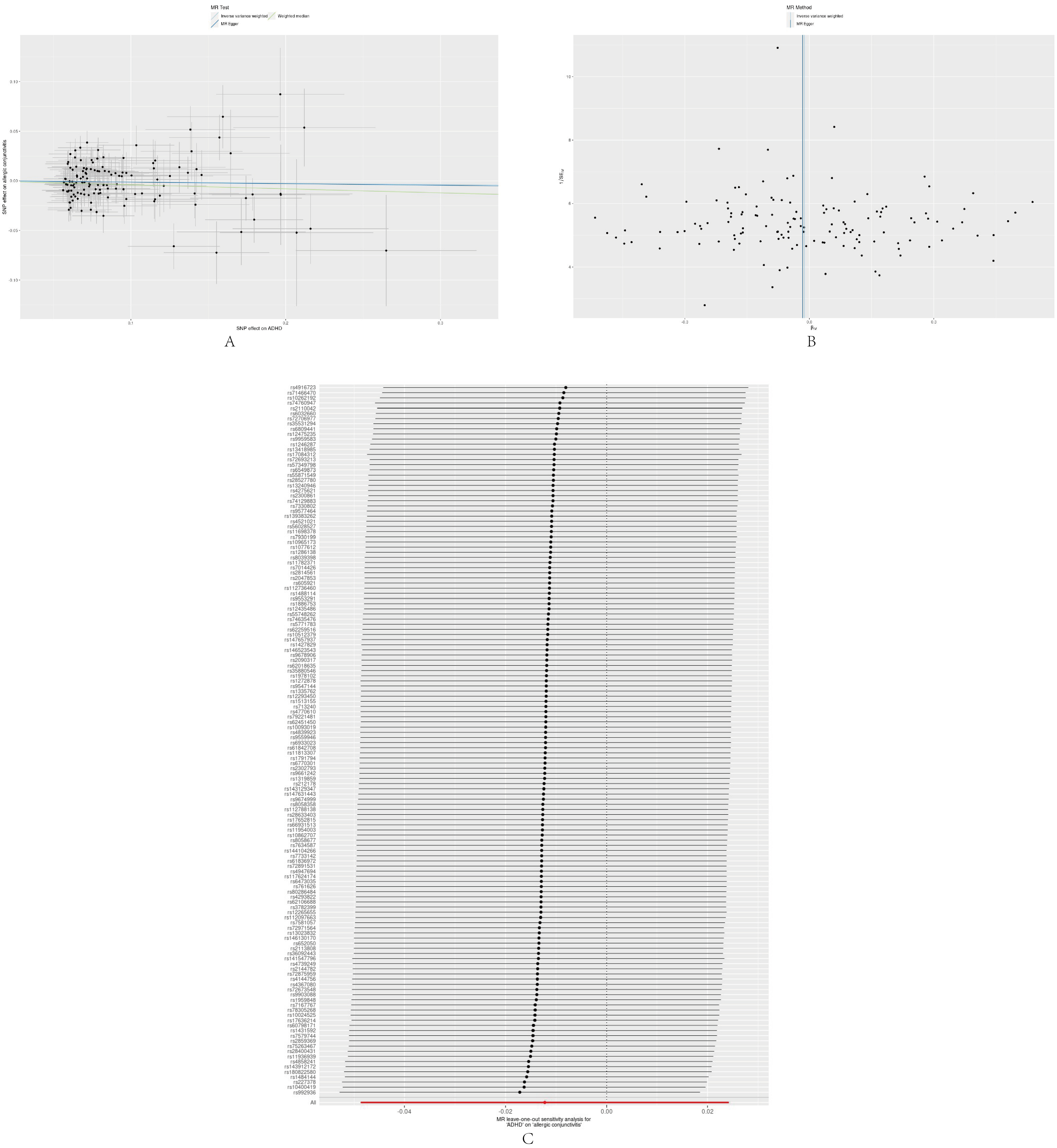

Supplement: Supplementary file 7 [file Image_4.JPEG]

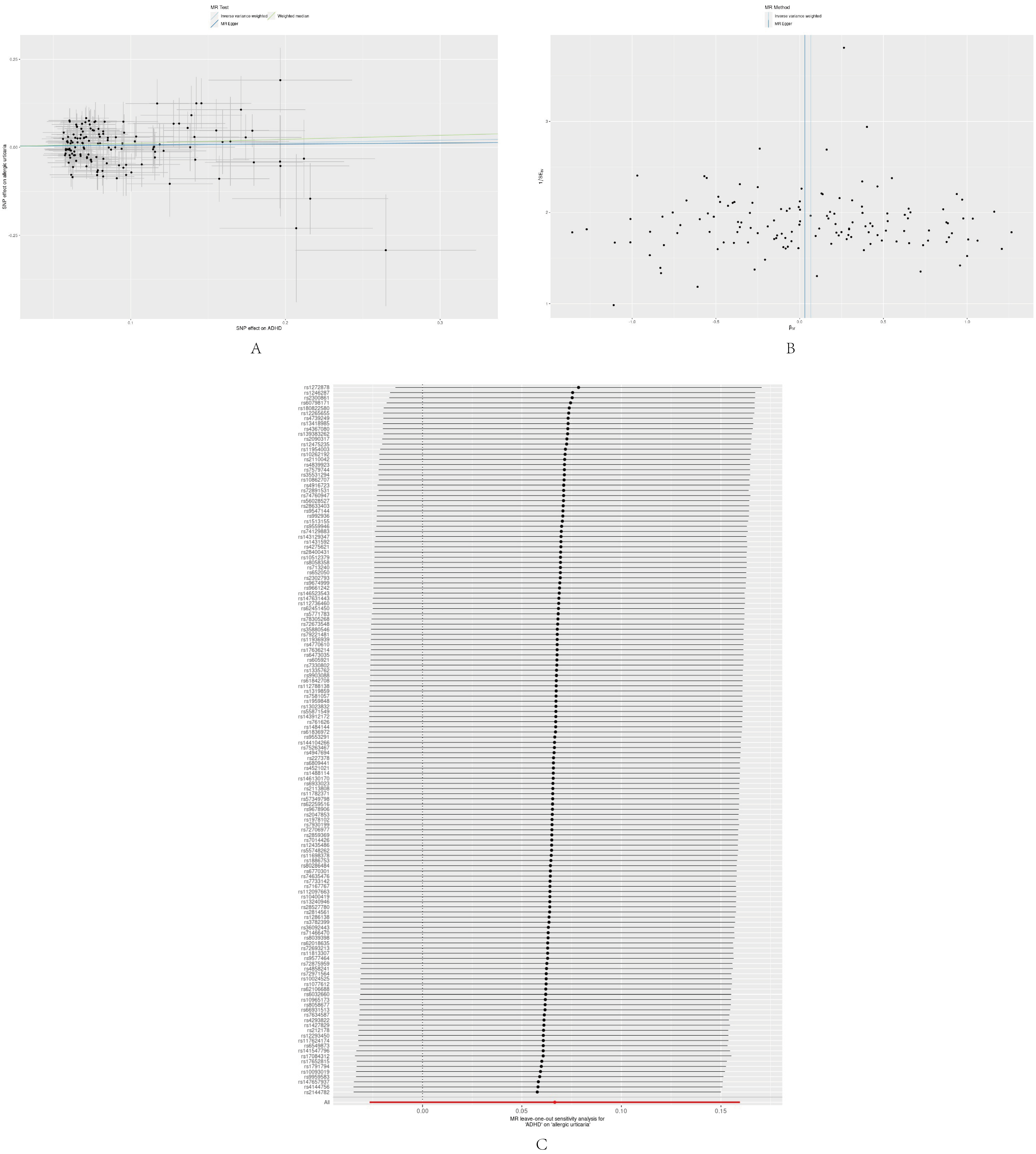

Supplement: Supplementary file 8 [file Image_5.JPEG]
